# What are we waiting for? The time to act on plastic pollution is now

**DOI:** 10.1016/j.lanwpc.2024.101107

**Published:** 2024-05-31

**Authors:** The Lancet Regional Health – Western Pacific

The reduction of plastic production was a contentious issue during the fourth session of the Intergovernmental Negotiating Committee on plastic pollution (INC-4) held in Ottawa, Canada, from April 23 to 29, 2024. The proposal to reduce primary plastic polymer production by 40% during 2025–40 was backed by several countries in the Western Pacific region, including Australia, Fiji, and the Philippines. In preparation for the final session (INC-5) scheduled for November 25–December 1, 2024 in Busan, South Korea, intersessional work will focus on implementation and funding of a global treaty on plastic pollution.

Plastic can be found everywhere and anywhere, from oceans to the foods we eat. Microplastics (less than 5 mm in diameter) and nanoplastics (less than 1 μm in diameter; MNPs) are tiny fragments of plastic, which can enter the body via ingestion (eg, water and seafood exposed to microplastics in the ocean) or inhalation (eg, cosmetics and personal care products and during laundering). There is growing evidence regarding the link between nanoparticles and health, but overall, the harmful effects of MNPs on human health remain incipient. In vivo studies suggest the harmful effects of MNPs on different organs as well as the digestive, immune, endocrine, and circulatory systems. Indeed, emerging evidence indicates a greater risk of myocardial infarction, stroke, and death among patients who had evidence of MNPs in carotid artery plaques, compared with those in whom MNPs in plaques were not detected.

To effectively tackle plastic pollution, reducing plastic production is fundamental. Between 2000 and 2019, global plastic production doubled to 460 million tonnes, accounting for 3% of global greenhouse gases. 90% of emissions arise from the production stage, whereby chemicals derived mostly from fossil fuels are transformed into plastics. In 2021, approximately 52% of the world’s plastics were produced in the Asia–Pacific region, with China producing 32% of plastics. An area referred to as the Great Pacific Garbage Patch, discovered in 1997, is a build-up of marine debris extending from the west coast of North America to Japan. It contains the largest amount of ocean plastic waste in the world, with microplastics comprising 94% of the estimated 1·8 trillion pieces of plastic debris. Within the Western Pacific, less than 15% of plastics are recycled. The global production of plastics is one of the major factors contributing to climate change in the Pacific Islands, one of the regions most vulnerable to the effects of climate change.

Solutions to reduce hazardous chemicals in plastic production are a neglected issue. A circular economy for plastics, whereby plastics are sustainably produced, designed, used, re-used and recycled could reduce the amount of plastic ending up in the ocean by over 80%, save governments US$70 billion by 2040, and cut greenhouse gas emissions by 25%. The Minderoo–Monaco Commission on Plastics and Human Health, including representatives from Japan and Australia, concluded that the current levels of production, use, and disposal of plastics are unsustainable, and production in particular is the primary driver of major harms to the environment and human health via increased exposure to the chemicals in plastics. As such, the Commission recommended that the global plastics treaty places a cap on plastic production and extends conditions to not only microplastics and marine litter, but also to the chemicals in plastics. Recent efforts to tackle plastic production and consumption in the region include new legislation in Hong Kong, which bans the sale and distribution of Styrofoam products and single-use plastic cutlery to encourage businesses and people to use reusable rather than single-use plastics. Initiatives include the development of a new declaration on 3R (reduce, reuse and recycle) and Circular Economy in Asia and the Pacific, as well as the CSIRO Ending Plastic Waste mission which focuses on research and development of technologies to prevent waste, salvage resources, and optimise the value of end-of-life materials in Australia. According to the national circular economy roadmap for plastics, glass, paper, and tyres from the CSIRO, the plastics processing capacity in Australia needs to grow by 150% at a minimum to prevent previously exported plastic waste going to landfill. An estimated EUR 25 billion annually is needed to enable low-income and middle-income countries to improve waste management infrastructure. Representatives from the Pacific Islands have called for the inclusion of a specific process for plastic pollution remediation for small island developing states (SIDS). Samoa spokesperson Katenia Rasch stated that, “It is important that SIDS should not be disproportionately burdened by remediation activities, given that we are already disproportionately impacted by transboundary plastic pollution. Language on innovation and technology development for remediation could also be useful in this section.” Accordingly, transition to a circular economy must be tailored to the social and economic circumstances of each country.

Whilst research on the environmental and health effects of plastic pollution continues to grow, tackling current practices in plastic production, consumption and disposal must be prioritised and placed at the forefront of the regional health agenda. It is clear that plastic pollution represents one of the most pressing threats to the environment, health, and economy in the Western Pacific region. Collaborative efforts are urgently needed to address the existing impact of plastic pollution in the region and mitigate inevitable future harms with no action. Ahead of the final session of the Intergovernmental Negotiating Committee, intersectoral and multisectoral approaches are fundamental for local contextualisation of plastic production, use and disposal strategies that reflect the unique needs of the region.

